# FOXC1 modulates MYOC secretion through regulation of the exocytic proteins RAB3GAP1, RAB3GAP2 and SNAP25

**DOI:** 10.1371/journal.pone.0178518

**Published:** 2017-06-02

**Authors:** Alexandra Rasnitsyn, Lance Doucette, Morteza Seifi, Tim Footz, Vincent Raymond, Michael A. Walter

**Affiliations:** 1 Department of Medical Genetics, Faculty of Medicine and Dentistry, University of Alberta, Edmonton, Alberta, Canada; 2 Centre Hospitalier de l'Université Laval (CHUL) Quebec City, Québec, Canada; Feinberg Cardiovascular Research Institute, Northwestern University, UNITED STATES

## Abstract

The neurodegenerative disease glaucoma is one of the leading causes of blindness in the world. Glaucoma is characterized by progressive visual field loss caused by retinal ganglion cell (RGC) death. Both surgical glaucoma treatments and medications are available, however, they only halt glaucoma progression and are unable to reverse damage. Furthermore, many patients do not respond well to treatments. It is therefore important to better understand the mechanisms involved in glaucoma pathogenesis. Patients with Axenfeld-Rieger syndrome (ARS) offer important insight into glaucoma progression. ARS patients are at 50% risk of developing early onset glaucoma and respond poorly to treatments, even when surgical treatments are combined with medications. Mutations in the transcription factor FOXC1 cause ARS. Alterations in FOXC1 levels cause ocular malformations and disrupt stress response in ocular tissues, thereby contributing to glaucoma progression. In this study, using biochemical and molecular techniques, we show that FOXC1 regulates the expression of RAB3GAP1, RAB3GAP2 and SNAP25, three genes with central roles in both exocytosis and endocytosis, responsible for extracellular trafficking. FOXC1 positively regulates RAB3GAP1 and RAB3GAP2, while either increase or decrease in FOXC1 levels beyond its normal range results in decreased SNAP25. In addition, we found that FOXC1 regulation of RAB3GAP1, RAB3GAP2 and SNAP25 affects secretion of Myocilin (MYOC), a protein associated with juvenile onset glaucoma and steroid-induced glaucoma. The present work reveals that FOXC1 is an important regulator of exocytosis and establishes a new link between FOXC1 and MYOC-associated glaucoma.

## Introduction

Glaucoma is among the leading causes of blindness in the world, affecting over 60 million people worldwide and is estimated to impact over 110 million people by 2040 [1]. Glaucoma often affects both eyes, however the damage tends to be asymmetric, affecting one of the eyes to a greater extent than the other [2]. Glaucoma is characterized by progressive visual field loss, caused by retinal ganglion cell (RGC) death [3]. The most common form of glaucoma is primary open angle glaucoma (POAG), and it has the highest prevalence in Africa where it affects 4.20% of the population between 40–80 years of age [1]. Current glaucoma treatments aim to halt glaucoma progression by lowering intraocular pressure (IOP), the buildup of aqueous flow resistance which contributes to RGC damage, but are unable to reverse damage [4,5]. In many cases, despite interventions, glaucomatous damage worsens with time, as ~ 27% of patients with open angle glaucoma may still face blindness in one eye, and 7% in both eyes after years of treatment [6]. Therefore, there is a need for better understanding of the pathogenic mechanisms causing glaucoma to develop novel therapies.

Axenfeld Rieger syndrome (ARS) is a part of the anterior segment dysgenesis spectrum of disorders [7]. Ocular malformations in ARS patients contribute to obstruction of aqueous humor outflow leading to increased IOP [8]. Patients with ARS are at 50% or greater risk of developing POAG, and in the majority of cases glaucoma develops between infancy and early adulthood [8,9]. Moreover, glaucoma in ARS patients is difficult to treat as both IOP lowering surgeries and medications have limited effect in halting glaucoma; only 18% of ARS patients respond to treatments, even when surgery and medication are combined [10]. ARS has an autosomal dominant inheritance and mutations in two developmental transcription factors, *PITX2* and *FOXC1*, are associated with ARS [11,12]. PITX2 regulates transcription of downstream genes by binding to upstream regions through its homeodomain and is involved in development of anterior segment tissues during embryogenesis [13]. FOXC1 and PITX2 are co-expressed in the mouse periocular mesenchyme and PITX2 interacts through its homeodomain with FOXC1 [14].

FOXC1 is a member of the Forkhead box (FOX) family of transcription factors, containing a conserved 110 amino acid Forkhead domain used to bind upstream of genes and promote gene activation [13]. Pathogenic *FOXC1* mutations include missense mutations within the Forkhead domain and nonsense and frameshift mutations in the upstream *FOXC1* region that result in a truncated protein [15,16]. Interestingly, some ARS patients have chromosomal duplications of *FOXC1* [15,17,18]. We previously conducted a microarray experiment in non-pigmented ciliary epithelium cells (NPCE) that identified genes whose expression changed following FOXC1 induction, among them several genes involved in exocytosis, responsible for extracellular trafficking [19]. Exocytosis is responsible for release of secretory molecules by both neuronal and non-neuronal cells [20]. Interestingly, exocytosis has an important role in glutamate excitotoxicity and other mechanisms involved in glaucoma [19,21,22]. Among the predicted FOXC1 exocytosis related gene targets were *RAB3GAP1* and *SNAP25* [19].

The predicted FOXC1 target, RAB3GAP1, with its sister protein RAB3GAP2, compose the heterodimeric complex RAB3GAP. RAB3GAP accelerates RAB3-GTP hydrolysis and promotes RAB3 to return to an inactive, GDP bound, state together with RAB3GEP, through interaction with its switch region [23]. RAB3 is important in secretory vesicle tethering and docking to the plasma membrane during exocytosis [24,25]. SNAP25 is a member of the SNARE complex responsible for fusion of vesicles with the plasma membrane during exocytosis [24]. To study the potential connection of FOXC1 to exocytosis, we validated the role of FOXC1 in RAB3GAP1 regulation and investigated regulation of RAB3GAP2 and SNAP25 by FOXC1. We found that FOXC1 positively regulates RAB3GAP1 and RAB3GAP2 and that SNAP25 regulation by FOXC1 is bimodular, where both decrease and increase in FOXC1 beyond its normal range decrease SNAP25 protein levels. In the trabecular meshwork (TM), the tissue through which the majority of aqueous humor drains into Schlemm’s canal, exocytosis is associated with MYOC secretion and ATP release. MYOC is a secreted protein associated with juvenile onset POAG (JOAG), referring to POAG diagnosed in patients between 2 and 40 years of age [26,27]. *MYOC* mutations were reported in 36% of JOAG patients [28]. As both, *FOXC1* and *MYOC*, are central genes in early-onset glaucoma pathogenesis we examined the interaction between these two genes, by looking at the effect of FOXC1 on exogenous MYOC secretion. We found that *FOXC1* knockdown, through regulation of *RAB3GAP1*, *RAB3GAP2* and *SNAP25*, leads to decrease in both intracellular and extracellular levels of MYOC, tying these genes into a common pathway of glaucoma pathogenesis.

## Results

### Enrichment of FOXC1 at upstream regions of *RAB3GAP1*, *RAB3GAP2* and *SNAP25*

**Fig 1:**
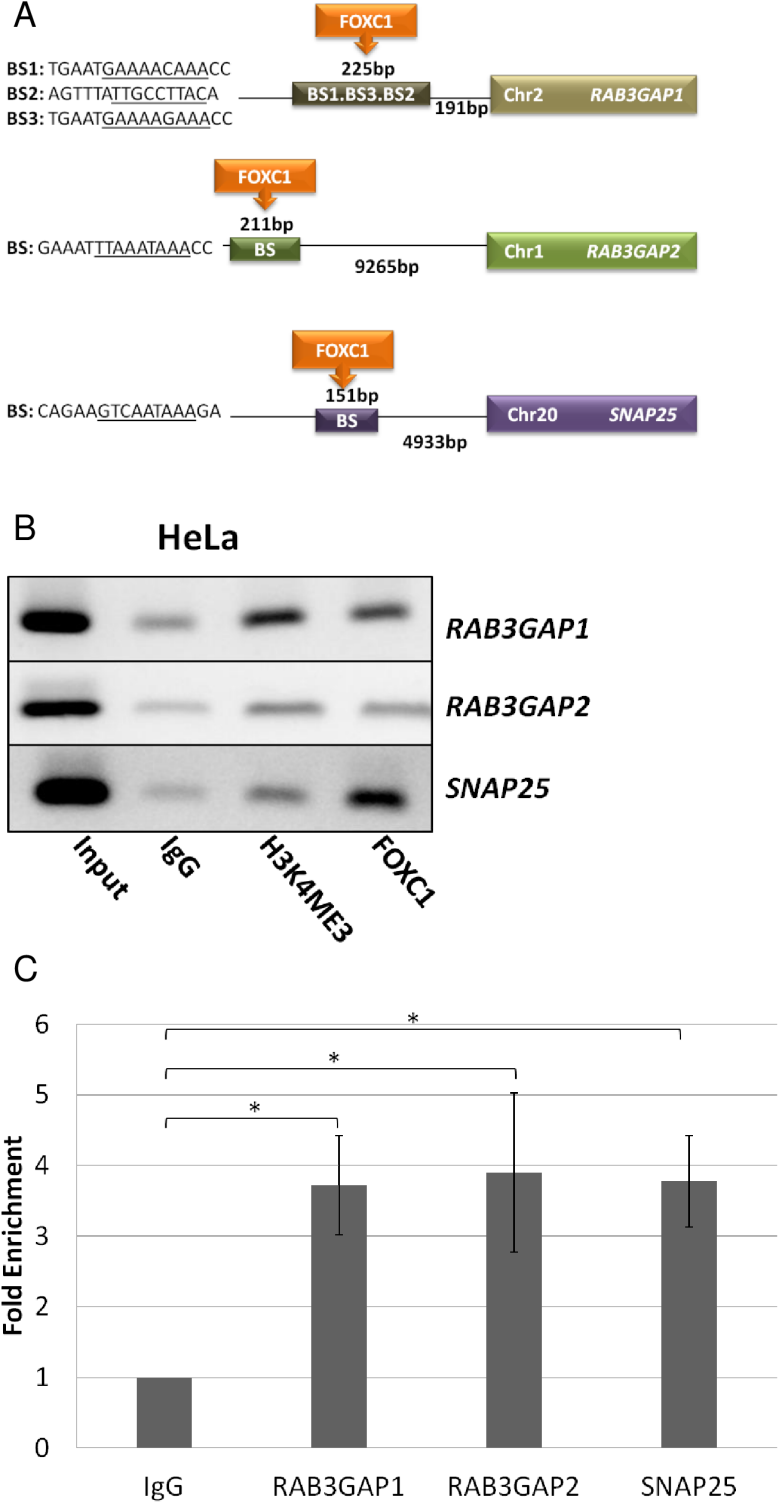
Enrichment of FOXC1 at upstream regions of *RAB3GAP1*, *RAB3GAP2* and *SNAP25*. **(A)** Potential FOXC1 binding sites found upstream of *RAB3GAP1*, *RAB3GAP2* and *SNAP25*. The predicted FOXC1 binding site is underlined. **(B)** Agarose gel electrophoresis of HeLa chromatin immunoprecipitated with antibodies for IgG (negative control), H3K4ME3 (active-chromatin marker) and FOXC1. Input sample represents cross-linked but not immunoprecipitated chromatin. **C)** qPCR results from IgG and FOXC1 immunoprecipitated chromatin for RAB3GAP1, RAB3GAP2, and SNAP25. *P = 0.05.

*RAB3GAP1*, *RAB3GAP2* and *SNAP25* were examined as potential *FOXC1* target genes in cultured Hela cells. *In silico* analysis identified potential FOXC1 binding sites (BS) found 191 bp upstream of *RAB3GAP1*, 9265 bp upstream of *RAB3GAP2*, and 4933 bp upstream of *SNAP25* (Fig 1A) using a FOXC1 DNA BS matrix and the Possum software. Chromatin immunoprecipitation (ChIP) assays were used to examine these FOXC1-binding sites upstream of *RAB3GAP1*, *RAB3GAP2* and *SNAP25*. ChIP analyses revealed enrichment of FOXC1 at the upstream regions of *RAB3GAP1*, *RAB3GAP2*, and *SNAP25* (Fig 1B), supporting interaction of FOXC1 with those regions. This result was confirmed in qPCR experiments which showed an overall enrichment of FOXC1 in upstream regions of *RAB3GAP1* (3.66 fold; P<0.05), *RAB3GAP2* (3.34 fold; P<0.05), and *SNAP25* (3.89 fold; P<0.05) compared to the mouse-IgG control (Fig 1C).

### Luciferase transactivation by FOXC1 through upstream regions of *RAB3GAP1*, *RAB3GAP2* and *SNAP25*

**Fig 2:**
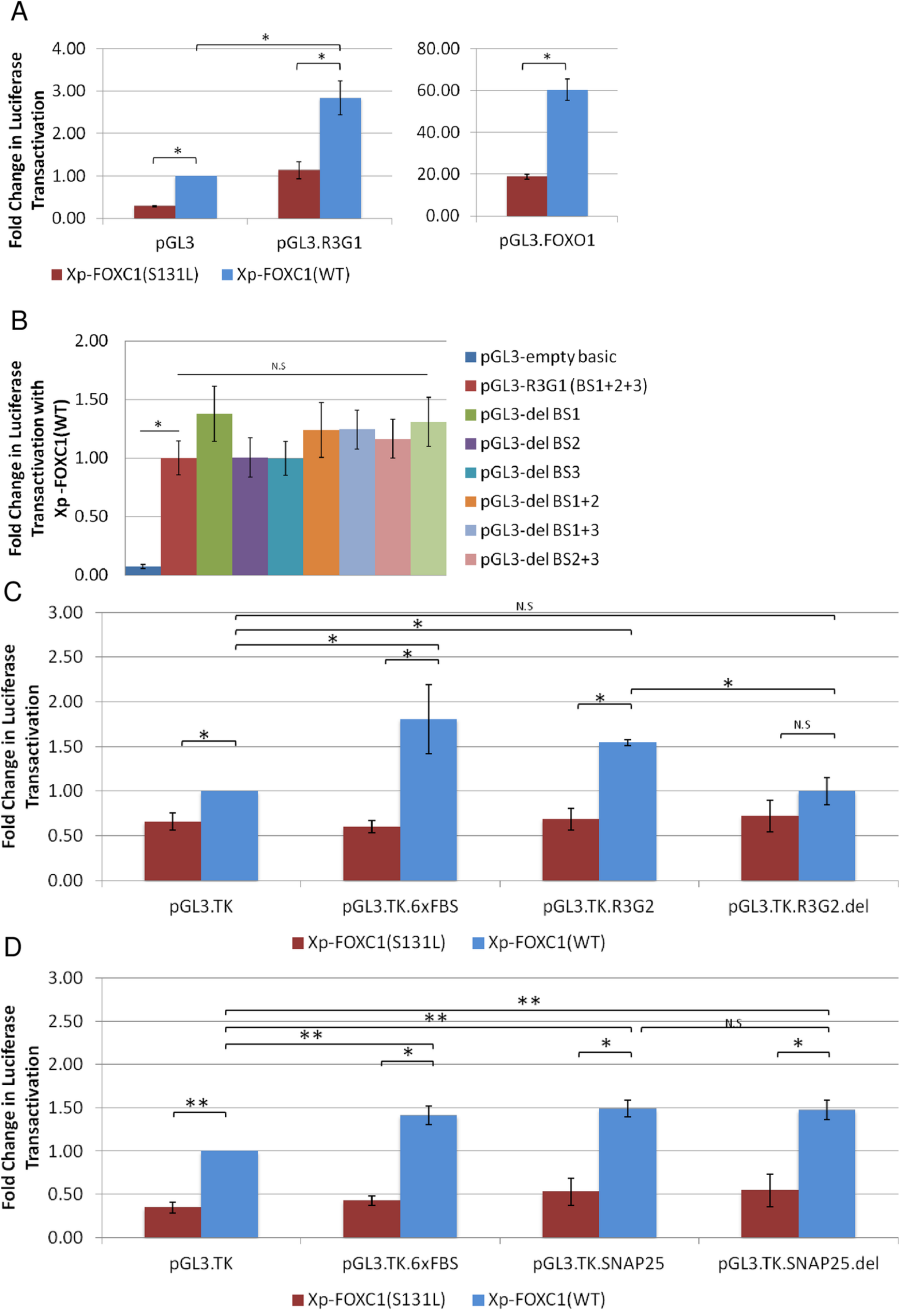
Luciferase transactivation by FOXC1 through upstream regions of *RAB3GAP1*, *RAB3GAP2* and *SNAP25*. Transactivation experiments with plasmid expressing FOXC1 (WT) or mutant FOXC1(p.S131L), and reporter construct (pGL3 or pGL3.TK) containing **(A)** The 225bp *RAB3GAP1* upstream (pGL3.R3G1) and pGL3.FOXO1 (positive control) **(B)** Upstream region of RAB3GAP1 (pGL3.R3G1), and constructs with each possible combination of putative FOXC1 binding sites deleted. **(C)** The 211bp *RAB3GAP2* upstream region, the region with the BS deleted, pGL3.TK.R3G2.del and pGL3.TK.6xFBS (positive control) **(D)** The 151bp *SNAP25* upstream region (pGL3.TK. SNAP25), and the region with the BS deleted (SNAP25.del). All Experiments were repeated at least three times in triplicate. Error bars represent standard error. N.S Not significant, *P˂0.05, **P˂0.01 versus pGL3.TK or pGL3.

To determine if FOXC1 could potentially activate the identified regions observed in the ChIP experiments, these regions were cloned independently into pGL3 luciferase reporter plasmids. The RAB3GAP1 upstream region contained three predicted binding sites (Fig 1A), BS1, BS2, and BS3. When HeLa cells were transfected with pGL3.R3G1, luciferase activation by FOXC1 (WT) significantly increased 2.8 fold compared transactivation observed with an empty pGL3 reporter (P< 0.05; Fig 2A). Additionally, when the pGL3.R3G1 construct was co-transfected with Xpress FOXC1 (WT), luciferase activity significantly increased 2.5 fold (P<0.05; Fig 2A) compared to co-transfection with Xpress FOXC1 (p.S131L), a FOXC1 mutant with reduced binding ability. We created constructs with each of the *RAB3GAP1* FOXC1 binding sites deleted individually, and in all possible combinations (pGL3-Del BS1, pGL3-Del BS2, pGL3-Del BS3, pGL3-Del BS1+2, pGL3-Del BS1+3, pGL3-Del BS2+3, pGL3-Del1+2+3). Transactivation assays using these deletion constructs showed no statistically significant differences from that of the pGL3.R3G1 construct (Fig 2B).

When HeLa cells were transfected with pGL3.R3G2 there was decrease in activation (S1 Fig). However, the sequence is located nearly 10 kb away from the transcription start site (Fig 1A). Therefore, it is likely that this potential FOXC1 binding site is in an enhancer region rather than a promoter element. To test this possibility, the *RAB3GAP2* upstream region and the upstream region with the predicted binding site deleted, were cloned into a pGL3.TK reporter (pGL3.TK.R3G2 and pGL3.TK.R3G2del), containing a basal promoter. Luciferase activity significantly increased 1.5 fold (P<0.05; Fig 2C) when pGL3.R3G2 was co-transfected with pcDNA4:Xpress FOXC1 (WT). In addition, there was a significant 2.5 fold increase (P<0.05; Fig 2C) in activation when pGL3.R3G2 was co-transfected with Xpress FOXC1 (WT) compared to co-transfection with Xpress FOXC1 (p.S131L), indicating the need for functional FOXC1 to promote activation. No difference was seen when cells were transfected with the deletion construct (pGL3.TK.R3G2.del) compared to the reporter (Fig 1C), indicating the importance of the predicted BS for activation.

A decrease in luciferase activation was also observed when pGL3.SNAP25 was co-transfected with pcDNA4:Xpress FOXC1 (WT) (S1 Fig). To test the possibility that this region may act as an enhancer, the 151 bp upstream region and the upstream region with the 16 bp predicted binding site (Fig 1A) removed were cloned into the pGL3.TK construct. Co-transfection of pGL3.TK.SNAP25 with Xpress FOXC1 (WT), resulted in a significant 1.5 fold increase in luciferase activation (P<0.05, Fig 2D), as compared to pGL3.TK. The Xpress FOXC1 (p.S131L) mutant construct was unable to activate luciferase expression (Fig 2D).

### *FOXC1* knockdown decreases RNA levels of *RAB3GAP1*, *RAB3GAP2* and *SNAP25*

**Fig 3:**
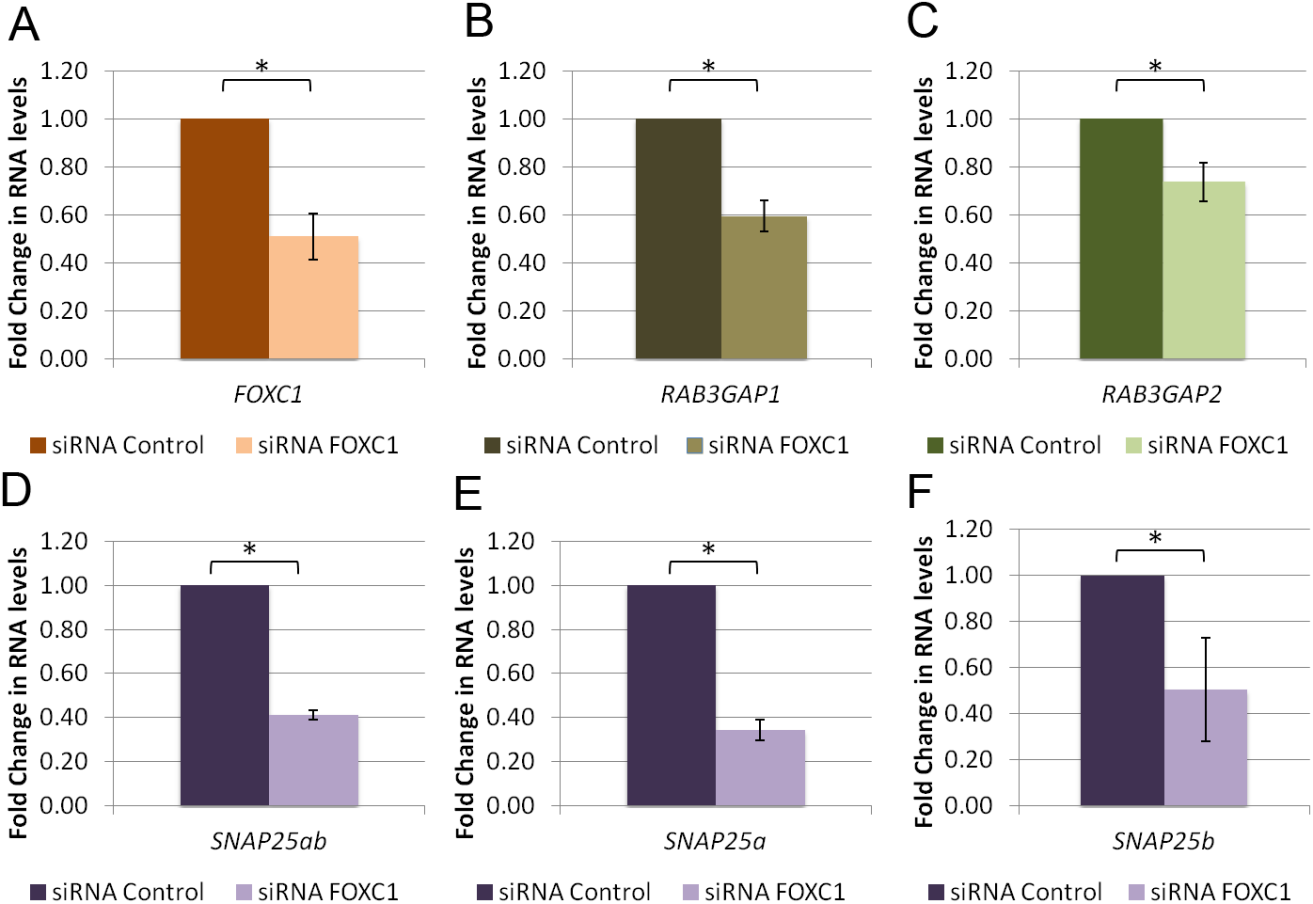
*FOXC1* knockdown decreases RNA levels of *RAB3GAP1*, *RAB3GAP2* and *SNAP25*. qRT-PCR experiments using RNA isolated from HeLa cells transfected with scrambled siRNA. qPCR was used to evaluate changes in RNA levels of **A)***FOXC1* **B)***RAB3GAP1* **C)***RAB3GAP2*, **D)** Total *SNAP25* (*SNAP25ab*) **E)** *SNAP25* isoform a (*SNAP25a*), **F)** *SNAP25* isoform b (*SNAP25b*) and *HPRT1* (housekeeping gene control). Fold change in RNA levels was calculated using the ΔΔCt method normalized to HPRT1 and scaled to siRNA scrambled control. Experiments were performed three times in triplicate. Error bars represent standard error. *P˂0.05.

qRT-PCR was used to determine if reducing FOXC1 levels would affect the RNA levels of *RAB3GAP1*, *RAB3GAP2*, and *SNAP25*. When *FOXC1* was significantly (P<0.05; Fig 3A) knocked down with siRNAs in HeLa cells, RNA levels of both *RAB3GAP1* and *RAB3GAP2* significantly (P<0.05; Fig 3B and 3C) decreased to 0.6 fold and 0.7 fold, respectively. Knockdown of *FOXC1* also significantly (P<0.05; Fig 3D–3F) reduced RNA levels of *SNAP25ab* to 0.4 fold, *SNAP25a* to 0.3 fold and *SNAP25b* to 0.5 fold.

### FOXC1 knockdown changes protein levels of RAB3GAP1, RAB3GAP2 and SNAP25

**Fig 4:**
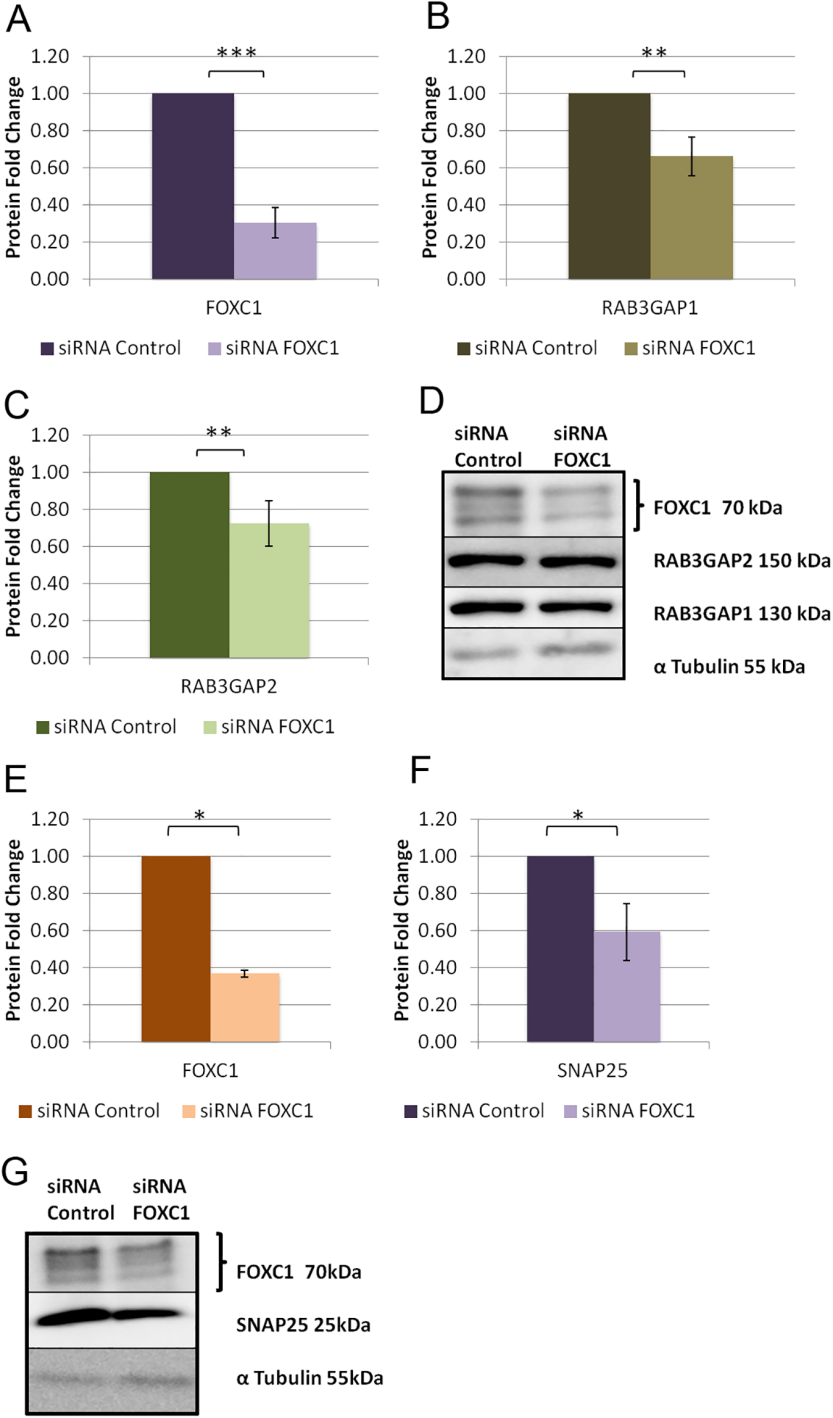
FOXC1 knockdown decreases protein levels of RAB3GAP1, RAB3GAP2 and SNAP25 in HeLa cells. **A)** Western blot analysis of HeLa protein lysates after siRNA transfection with siRNA Scrambled control, or siRNA FOXC1. As FOXC1 has a number of phosphorylation sites, a number of bands are observed at ~70kDa, indicated by a curly brace. Blots were probed for FOXC1, RAB3GAP1, RAB3GAP2, SNAP25 and α-Tubulin (loading control). Normalized and scaled values for **B)** RAB3GAP1 **C)** RAB3GAP2 and **D)** SNAP25. All Western blots were repeated at least three times. *P˂0.05, **P˂0.01, ***P<0.001.

Western blot analysis was used to determine the effect of FOXC1 knockdown on target genes protein levels. When FOXC1 was significantly (P<0.001; Fig 4A) knocked down using siRNA’s in HeLa cells to 0.3 fold, a significant (P<0.01; Fig 4B and 4C) decrease in protein levels of both RAB3GAP1 and RAB3GAP2 was observed to 0.7 fold. In turn, SNAP25 protein levels significantly (P<0.05; Fig 4F) decreased to 0.6 fold when FOXC1 was significantly (P<0.05; Fig 4E) knocked down to 0.4 fold supporting positive regulation by FOXC1.

### FOXC1 over-expression changes protein levels of RAB3GAP1, RAB3GAP2 and SNAP25

**Fig 5:**
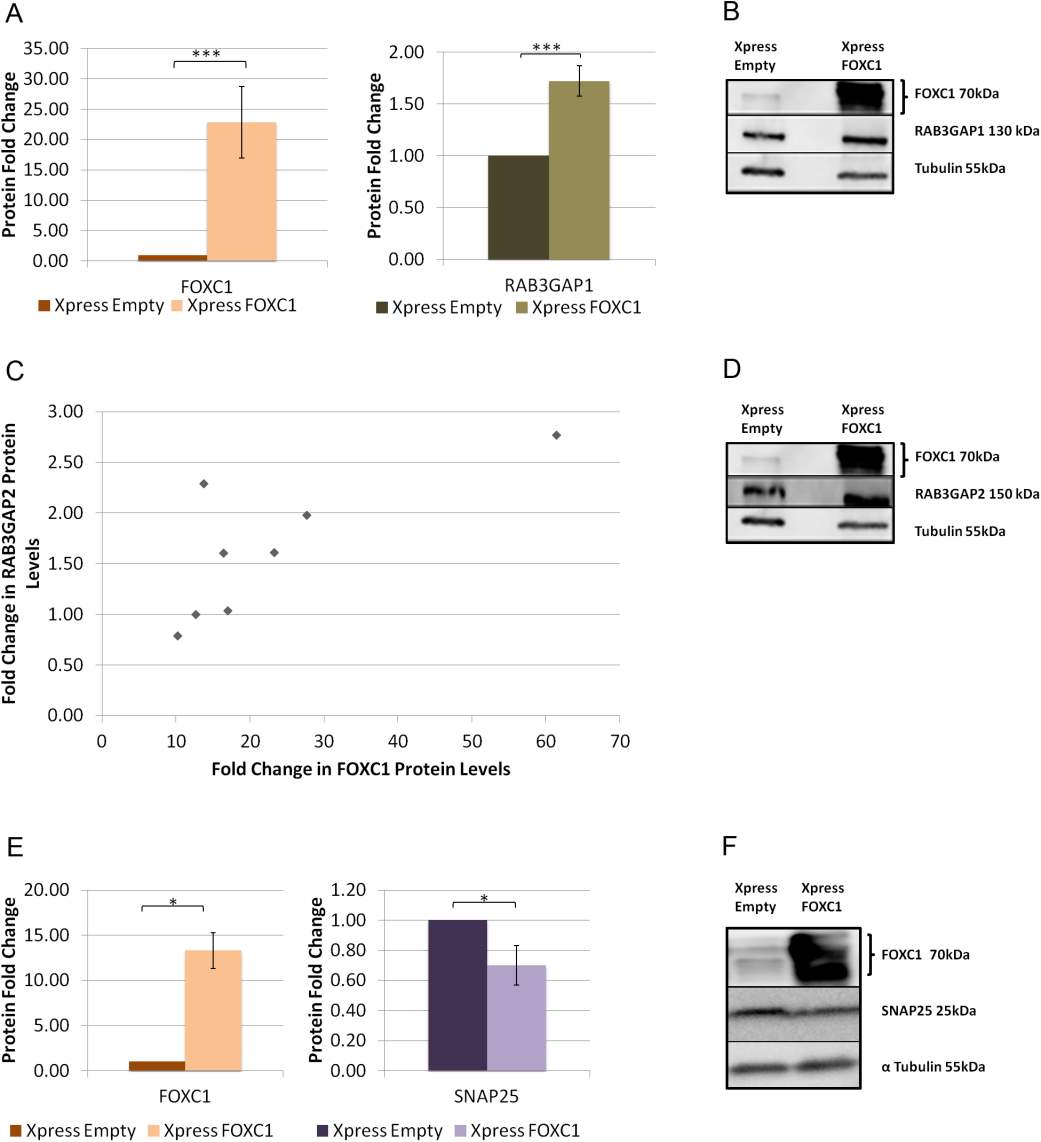
FOXC1 over-expression changes protein levels of RAB3GAP1, RAN3GAP2 and SNAP25 in HeLa cells. Western blot analysis of HeLa protein lysates after transfection with either pcDNA4-Xpress-Empty or pcDNA4-Xpress-FOXC1(WT). Blots were probed with antibodies for FOXC1, RAB3GAP1, RAB3GAP2, SNAP25 and α-Tubulin (loading control). Normalized scaled values were calculated for **A)** RAB3GAP1 (example blot shown in **B**). Normalized and scaled values compared to level of FOXC1 knockdown are presented in **C)** RAB3GAP2 (example blot shown in **D**) and **E)** SNAP25 (example blot shown in **F**) *P˂0.05. Experiments were repeated at least three times.

The effect of FOXC1 over-expression on target gene protein levels was also investigated. When FOXC1 was significantly (P<0.001; Fig 5A) over-expressed, using a FOXC1 (WT) plasmid, 22.8 fold, protein levels of RAB3GAP1 significantly (P<0.001; Fig 5B) increased 1.7 fold. Changes in RAB3GAP2 protein levels following FOXC1 over-expression showed a more complicated pattern. RAB3GAP2 protein levels increased only when FOXC1 protein was over-expressed more than 13 fold (Fig 5C and 5D). However, overall, FOXC1 and RAB3GAP2 levels were positively (0.72), and significantly (P<0.05; Fig 5C) correlated in HeLa cells.

In contrast, SNAP25 protein levels significantly decreased to 0.7-fold (P<0.05; Fig 5F) when FOXC1 protein was significantly over-expressed 13.3 fold (P<0.05; Fig 5E).

### Knockdown of FOXC1 and FOXC1’s exocytotic protein targets RAB3GAP1, RAB3GAP2 and SNAP25 affects secretion of exogenous MYOC

To test the effect of FOXC1 and its target genes on secretion, MYOC assays were used. MYOC was expressed in HeLa cells using the plasmid pRcMYOC [29], and was detected both in whole cell protein extracts (bands at 57 kDa and 55 kDa representing the glycosylated and non-glycosylated forms respectively) and in the cell media (S2 Fig).

**Fig 6:**
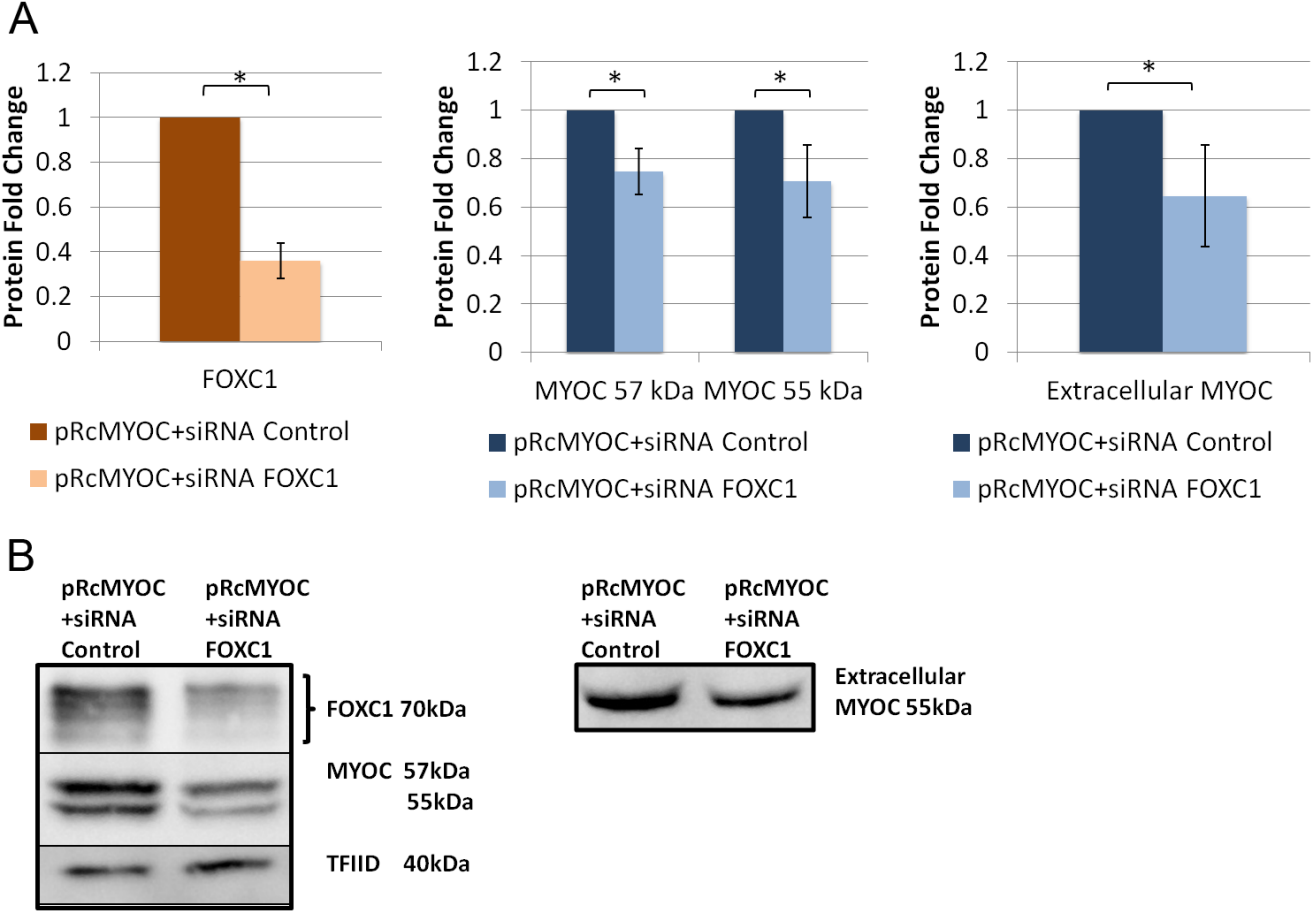
FOXC1 knockdown decreases intracellular and extracellular levels of exogenous MYOC in HeLa cells. Western blot analysis using HeLa cell lysate, and cell media collected after transfection with pRc-MYOC (WT) and either siRNA Control or siRNA FOXC1. Blots of lysates were probed and bands were quantified, normalized, and scaled for A) FOXC1, and MYOC using TFIID as a loading control and blots of media were probed for MYOC using ponceau stain intensity as a loading control (right). **B)** Example blots of lysates (left) and media (right). Error bars represent standard error. *P˂0.05.

**Fig 7:**
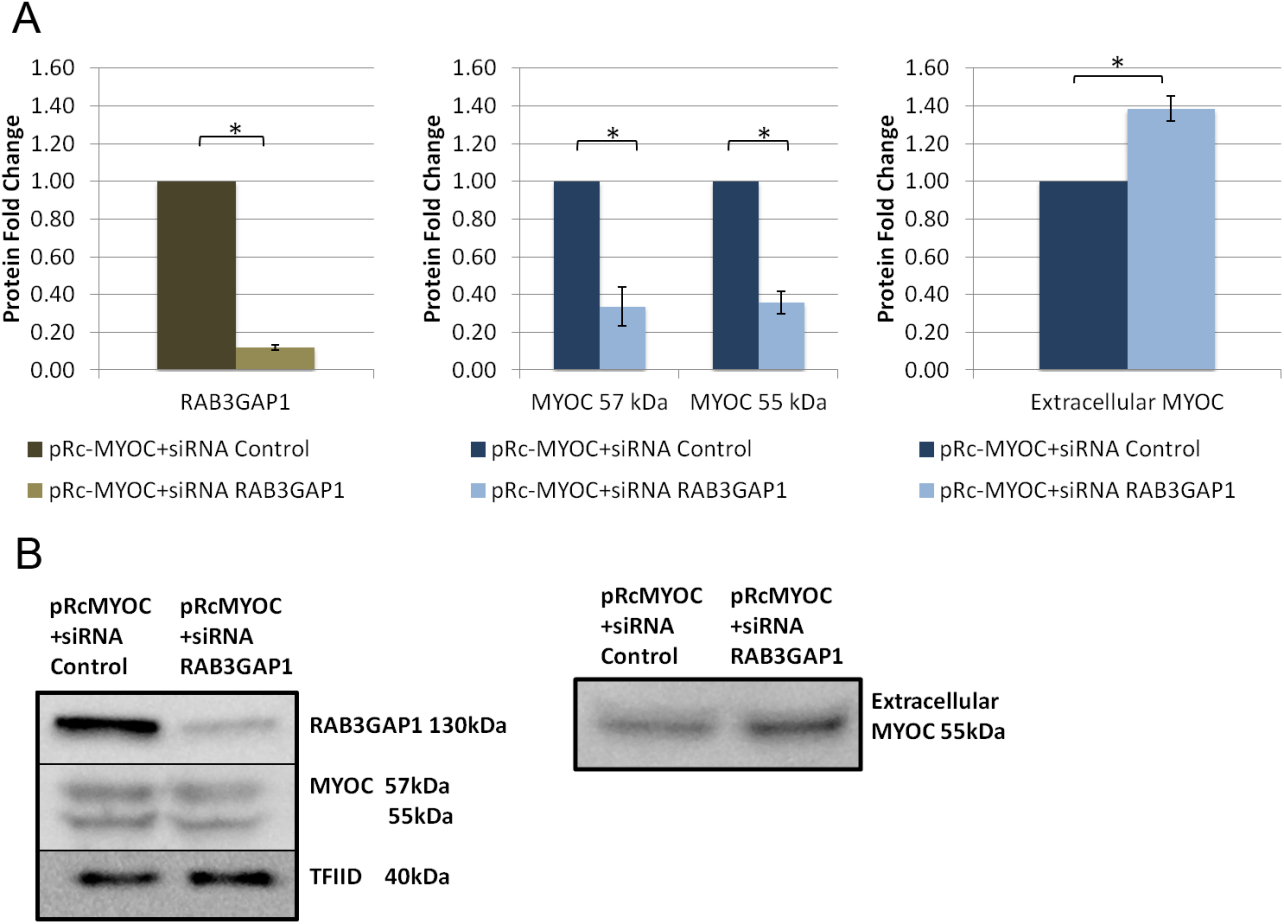
RAB3GAP1 knockdown decreases intracellular and increases extracellular levels of exogenous MYOC in HeLa cells. Western blot analysis using HeLa cell lysate, and cell media collected after transfection with pRc-MYOC (WT) and either siRNA Control or siRNA RAB3GAP1. Blots of lysates were probed and bands were quantified, normalized, and scaled for A) RAB3GAP1, and MYOC using TFIID as a loading control and blots of media were probed for MYOC using ponceau stain intensity as a loading control (right). **B)** Example blots of lysates (left) and media (right). Error bars represent standard error. *P˂0.05.

**Fig 8:**
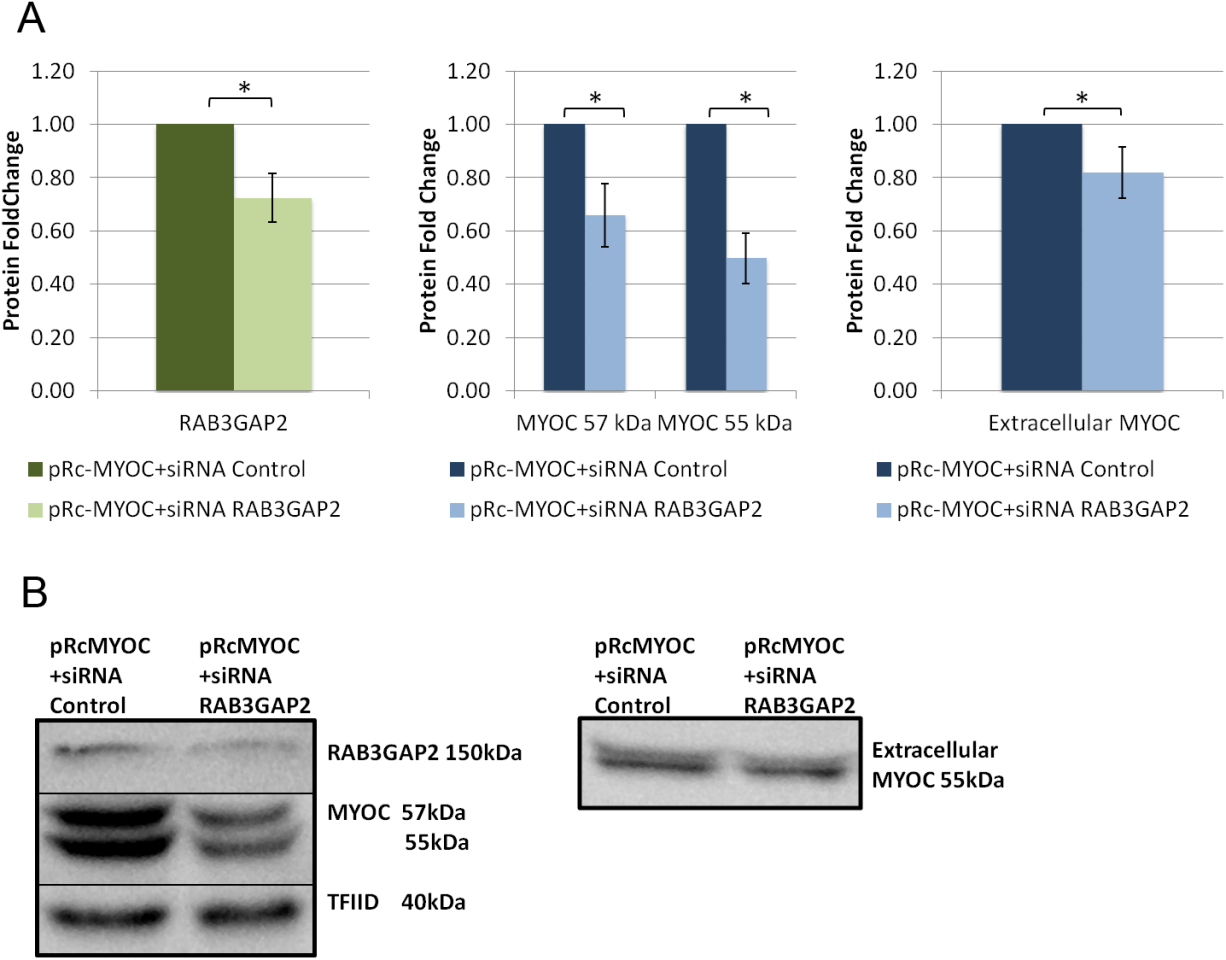
RAB3GAP2 knockdown decreases intracellular and extracellular levels of exogenous MYOC in HeLa cells. Western blot analysis using HeLa cell lysate, and cell media collected after transfection with pRc-MYOC (WT) and either siRNA Control or siRNA RAB3GAP1. Blots of lysates were probed and bands were quantified, normalized, and scaled for A) RAB3GAP2, and MYOC using TFIID as a loading control and blots of media were probed for MYOC using ponceau stain intensity as a loading control (right). **B)** Example blots of lysates (left) and media (right). Error bars represent standard error. *P˂0.05.

**Fig 9:**
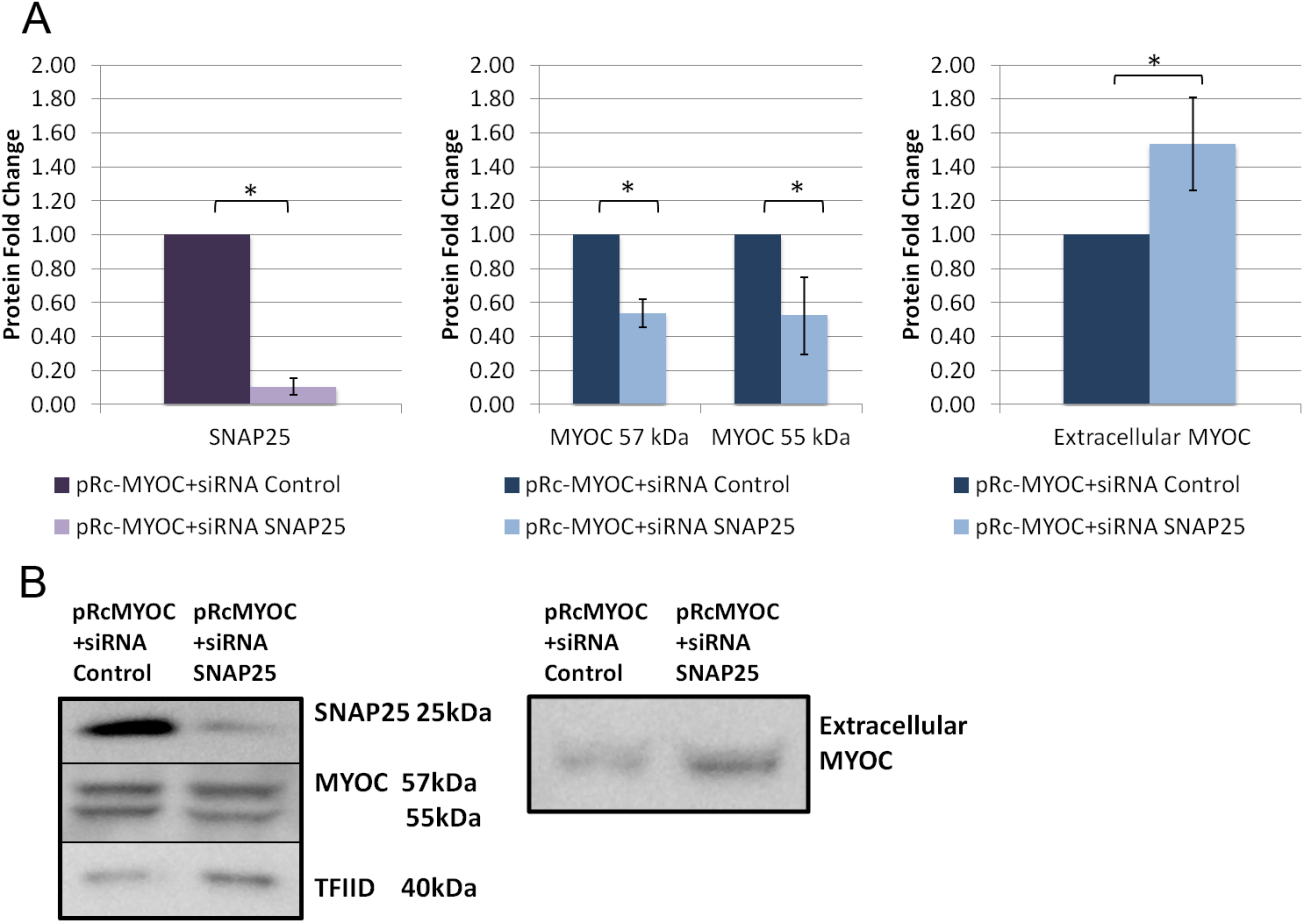
SNAP25 knockdown decreases intracellular and increases extracellular levels of exogenous MYOC in HeLa cells. Western blot analysis using HeLa cell lysate, and cell media collected after transfection with pRc-MYOC (WT) and either siRNA Control or siRNA RAB3GAP1. Blots of lysates were probed and bands were quantified, normalized, and scaled for A) SNAP25, and MYOC using TFIID as a loading control and blots of media were probed for MYOC using ponceau stain intensity as a loading control (right). **B)** Example blots of lysates (left) and media (right). Error bars represent standard error. *P˂0.05.

FOXC1 was knocked down in HeLa cells, using siRNAs, to evaluate its effect on exogenous MYOC secretion. When FOXC1 was significantly knocked down to 0.4 fold (P<0.05; Fig 6A), intracellular MYOC protein levels significantly (P<0.05; Fig 6A) decreased to 0.8 and 0.7 fold for the MYOC 57kDa and 55 kDa respectively, while extracellular MYOC protein levels decreased to 0.7 fold (P<0.05; Fig 6A). Next, we examined if the effect of FOXC1 on exogenous MYOC is through FOXC1 regulation of RAB3GAP1, RAB3GAP2 and SNAP25. MYOC secretion experiments were repeated with each of the target genes knocked down, with siRNAs, separately and in combinations when co-transfected with pRcMYOC. When RAB3GAP1 was significantly (P<0.05; Fig 7A) knocked down to 0.1 fold, MYOC protein levels significantly decreased to 0.3 fold at 57 kDa and 0.4 fold at 55 kDa (P<0.05; Fig 7A) and extracellular levels significantly increased 1.4 fold (P<0.05; Fig 7A). When RAB3GAP2 was significantly knocked down to 0.7 fold (P<0.05; Fig 8A) intracellular levels of MYOC significantly decreased to 0.7 fold (P<0.05; Fig 8A) at 57 kDa and 0.5 fold at 55 kDa and extracellular protein levels significantly decreased to 0.8 fold (P<0.05; Fig 8A). A similar effect on MYOC protein levels was observed when SNAP25 was knocked down. Significant decrease of SNAP25 to 0.1 fold (P<0.05; Fig 9A) led to a significant decrease in intercellular MYOC protein to 0.5 fold at both 57 kDa and 55 kDa (P<0.05; Fig 9A), and a significant 1.5 fold increase in extracellular MYOC protein (P<0.05; Fig 9A).

**Fig 10:**
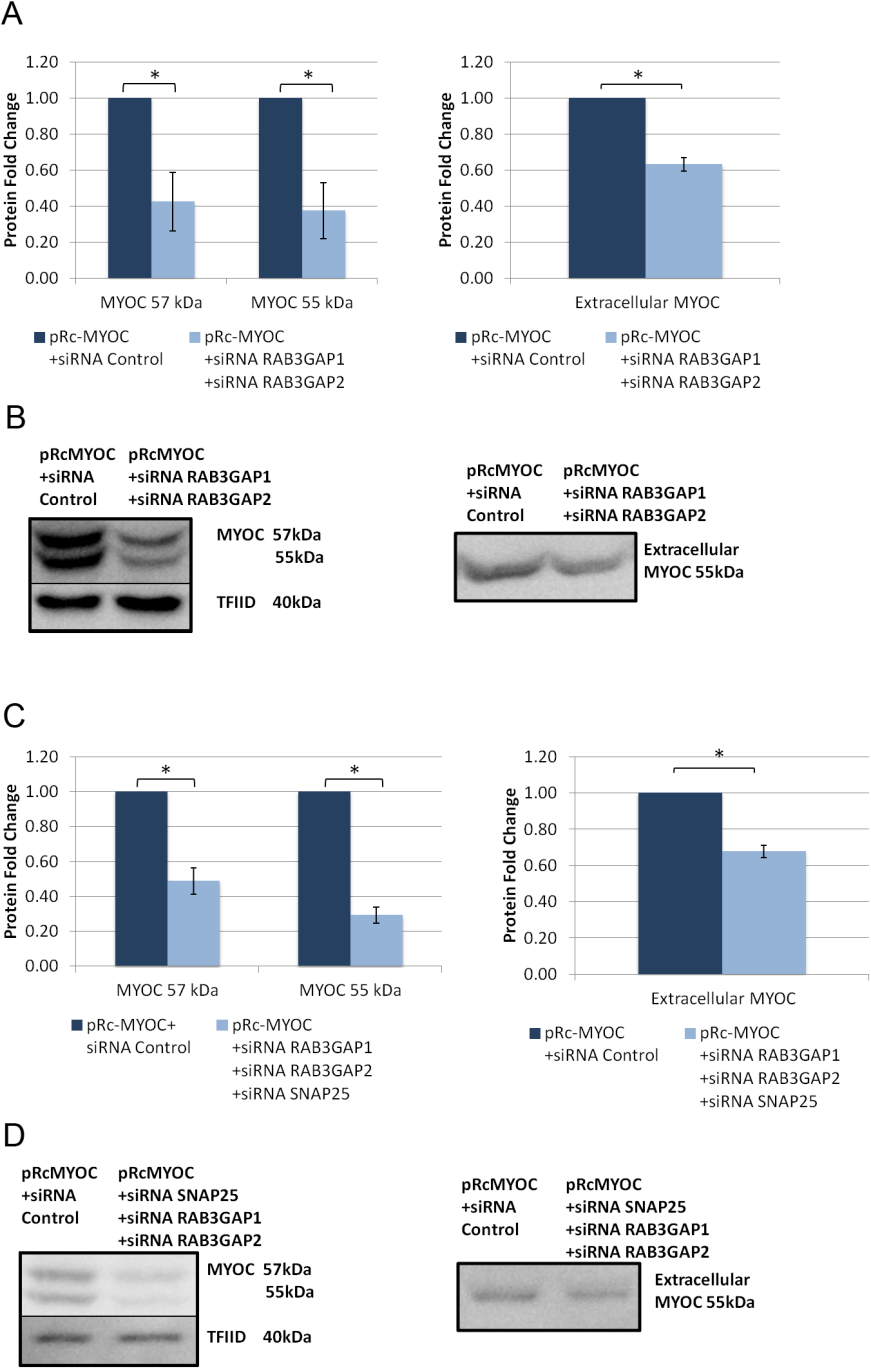
Combined RAB3GAP1, RAB3GAP2 and SNAP25 knockdown decreases intracellular and extracellular levels of exogenous MYOC in HeLa cells. **A)** Western blot analysis of intracellular (left) and extracellular (right) MYOC upon knockdown of RAB3GAP1 and RAB3GAP2 in HeLa cells transfected with pRc-MYOC(WT) **(B)** Example Western blot of A. **C)** Western blot analysis of intracellular (left) and extracellular (right) MYOC following SNAP25, RAB3GAP1 and RAB3GAP2 knockdown in HeLa cells transfected with pRc-MYOC(WT). **D)** Example Western blot of C.

**Fig 11:**
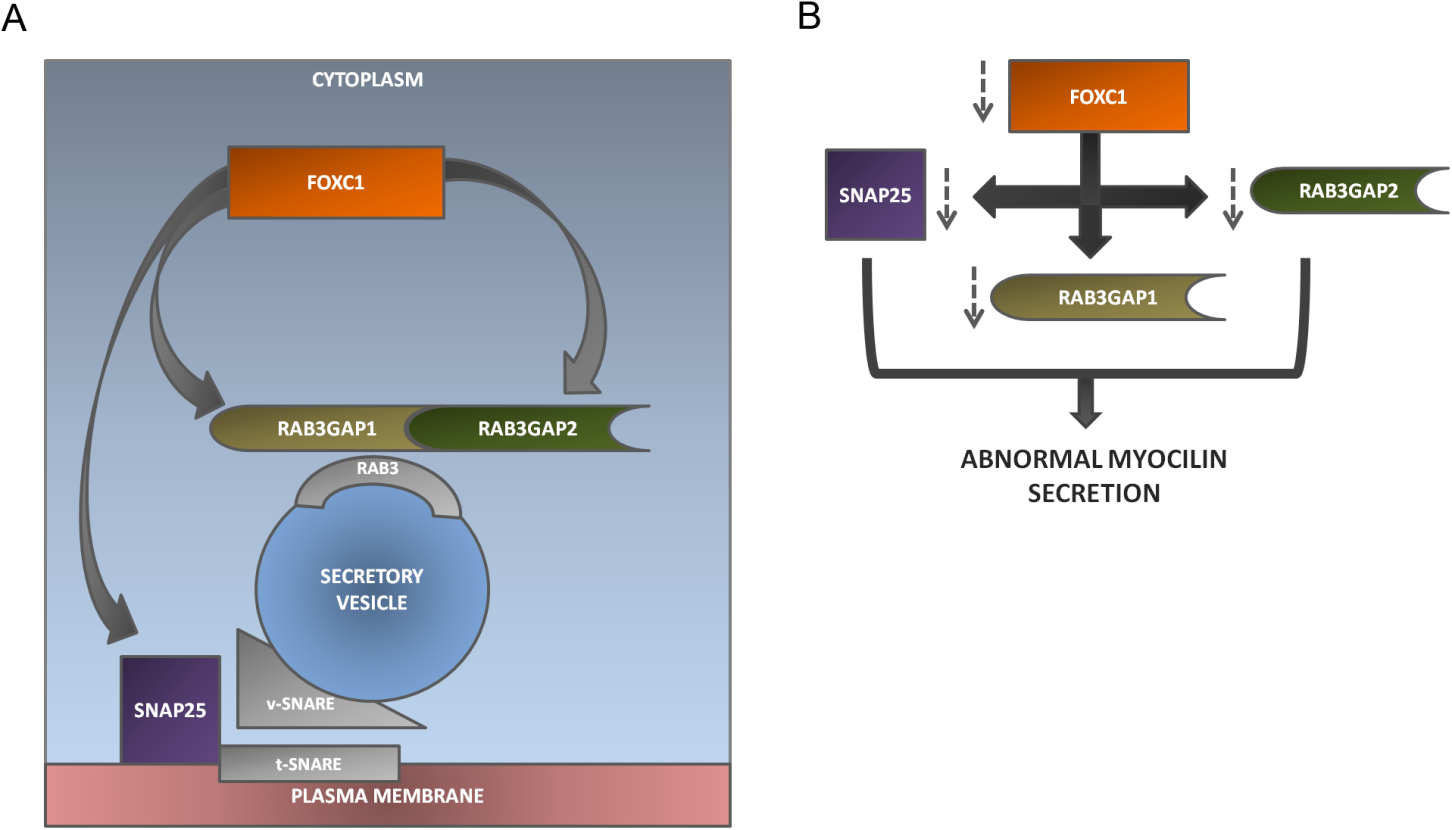
Summary of the regulation of RAB3GAP1, RAB3GAP2 and SNAP25 by FOXC1 and its effect on Myocilin secretion. **(A)** FOXC1 regulates RAB3GAP1 and RAB3GAP2 which compose the RAB3GAP complex involved in inactivation of RAB3, responsible for bringing secretory vesicles near the plasma membrane. FOXC1 also regulates SNAP25, a member of the SNARE complex, which during exocytosis forms a complex with another v-SNARE and t-SNARE thereby fusing the secretory vesicle with the plasma membrane. **(B)** FOXC1 knockdown leads to decreased protein levels of RAB3GAP1, RAB3GAP2 and SNAP25 which in turn results in abnormal Myocilin secretion.

As FOXC1 knockdown was shown to reduce protein levels of all three targets in HeLa cells, the target genes were knocked down simultaneously to simulate the effect of FOXC1. First, RAB3GAP1 and RAB3GAP2 were knocked down at the same time. When siRNAs against both genes were used, intracellular levels of MYOC protein significantly decreased to 0.4 fold (P<0.05; Fig 10A) at 57 kDa and 0.6 fold at 55 kDa and extracellular levels significantly decreased to 0.6 fold (P<0.05; Fig 10A). Knockdown of all three genes with siRNA resulted in significant decrease in intracellular MYOC to 0.5 fold at 57 kDa and 0.3 fold at 55 kDa (P<0.05; Fig 10C) and significant decrease in extracellular levels (P<0.05; Fig 10C) to 0.7 fold. Both combination treatments have a similar effect on MYOC secretion as FOXC1 knockdown, indicating that the effect of FOXC1 on MYOC secretion is exerted through regulation of RAB3GAP1, RAB3GAP2 and SNAP25 (Fig 11B).

## Discussion

ARS is an important model for glaucoma pathogenesis, due the heightened risk of ARS patients to develop early onset glaucoma and their lack of response to glaucoma treatments.[8–10] Mutations leading to abnormal *FOXC1* copy number cause ARS and Dandy-Walker syndrome, characterized by abnormal cerebellum development [12,30]. The involvement of FOXC1 in disorders with ocular and neurodevelopmental malformations and in the neurodegenerative disease glaucoma illustrates its importance in neural and ocular development and neuronal health.

In the present work FOXC1 is shown to regulate the exocytosis associated genes *RAB3GAP1*, *RAB3GAP2* and *SNAP25* and modulate MYOC secretion (Fig 11). The new connection to MYOC and exocytosis indicates FOXC1’s involvement in additional mechanisms related to glaucoma and further elucidates the mechanisms through which FOXC1 is involved in neurodevelopment and function.

*RAB3GAP1* and *SNAP25* were detected as potential targets of FOXC1 regulation in a microarray performed in NPCE cells [19]. FOXC1 knockdown was also shown to decrease *RAB3GAP1* RNA levels in ocular tissues of zebrafish and NPCE cells [19]. Our work further validates *RAB3GAP1* as a FOXC1 target. Our work has shown that FOXC1 can interact with the upstream region of *RAB3GAP1* and upregulates luciferase transactivation through this region illustrating the importance for FOXC1 binding to the upstream region for transactivation. However, deletion of any or all of the predicted FOXC1 binding sites from the upstream region of *RAP3GAP1* had no significant effect on luciferase transactivation. This suggests that FOXC1 may bind other sequences in this fragment not identified by *in silico* analyses, or it’s possible that it may be acting as a recruitment factor important in the initiation for transcription. It is also possible that FOXC1 is working with an unknown interacting accessory protein to bring about these effects, and that RAB3GAP1 is indirectly regulated by FOXC1. However, as the activation of FOXC1 on this upstream region is significantly higher than a mutant protein (p.S131L), it is evident that active FOXC1 is necessary for this transactivation to occur. In addition, we showed that FOXC1 positively regulates *RAB3GAP1* RNA and protein levels.

RAB3GAP1 composes RAB3GAP complex along with RAB3GAP2. RAB3GAP1 is the catalytic subunit of this complex, while RAB3GAP2 is the non-catalytic unit. As these two proteins are closely related, we hypothesized that FOXC1 may potentially regulate RAB3GAP2 as well. We showed that FOXC1 can interact with a *RAB3GAP2* upstream region predicted through *in silico* analyses. When the construct containing the RAB3GAP2 upstream region (pGL3:R3G2) was transfected into HeLa cells alone, no activation was seen. Given this data, and that the upstream region is 10kb away from the transcriptional start site, we hypothesized that this region may be an enhancer element. To test this possibility, we cloned this region upstream of a basal TK promoter. Transfection of the construct with Wild-Type FOXC1 increased luciferase transactivation, and to significantly greater extent than mutant FOXC1. Furthermore, deletion of the predicted FOXC1 binding site showed reduced luciferase transactivation, indicating that FOXC1 interacts with this upstream region through the 5’-GAAATTTAAATAAACC-3‘ sequence. FOXC1 was able to increase luciferase transactivation through the upstream *RAB3GAP2* region only in the presence of the TK promoter, supporting that the FOXC1 binding site is not located in a promoter region but rather in an enhancer region. Enhancer regions can be located far from the transcription start site (TSS) [31]. When enhancer regions are activated, for example by transcription factor binding, they can undergo chromatin remodeling to become closer to the promoter and boost transcription by helping the general transcription factors to assemble.[31] Based on this data, FOXC1 likely enhances *RAB3GAP2* transcription by recruiting other transcription factors or accessory proteins to interact with the promoter region rather than acting on it directly. We have also shown that FOXC1 knockdown decreased RNA and protein levels of *RAB3GAP2* while over-expression of FOXC1 correlated with increase of RAB3GAP2 protein levels. Taken together, these results indicate that FOXC1 positively regulates RAB3GAP2 expression.

The RAB3GAP complex is important in inactivating RAB3 by promoting its GTP hydrolysis [32]. In its activated form, RAB3 forms complexes with various effectors to direct progress of both exocytosis and endocytosis, responsible for extracellular trafficking [33,34]. However, RAB3 inactivation is necessary for it to dissociate from its effectors and repeat its action in the exocytosis-endocytosis cycle of cell trafficking [35]. We predict that FOXC1 regulates the RAB3 complex indirectly through its regulation of RAB3GAP1 and RAB3GAP2. RAB3 is involved in synaptic vesicle and granule secretion as well as regulated secretion [25]. Therefore, through regulation of RAB3GAP1 and RAB3GAP2, FOXC1 is predicted to influence the balance of active versus inactive RAB3. This provides an interesting hypothesis in terms of glaucoma pathogenesis. Exocytosis in glaucoma is involved in glutamate excitotoxicity, ATP secretion, and potentially MYOC secretion, indicating novel pathogenic mechanisms through which FOXC1 might promote glaucoma [36–38]. RAB3 was also shown to be involved in endocytosis, as its effecter Rabfilin-3 needs to be released following RAB3 inactivation to interact with Rabaptin-5 and promote receptor mediated transferrin endocytosis [38]. Endocytosis abnormalities in glaucoma are associated with *OPTN* mutations, shown to interrupt transferrin receptor recycling and promote autophagic death [39–41]. Autophagy is responsible for breakdown and transport of cytosolic components to lysosomes where they are degraded [42]. Therefore, it is possible that FOXC1 mutations affect RAB3 involvement in endocytosis and potentially act in similar pathogenic mechanisms to *OPTN* mutations. Overall, FOXC1 regulation of RAB3GAP opens new directions for FOXC1 involvement in glaucoma pathogenesis through regulations of exocytosis, endocytosis and autophagy.

Another exocytotic target detected in the microarray by Berry et al. (2008) was *SNAP25* [19]. SNAP25 plays a central role in secretory vesicle fusion with the plasma membrane through formation of the SNARE complex. SNAP25 was predicted to be negatively regulated in NPCE cells by FOXC1. This is an interesting result as FOXC1 is typically known as a transactivator rather than a repressor [19]. FOXC1 interacted with the upstream region of SNAP25 and increased luciferase transactivation when the SNAP25 upstream region was cloned upstream of a TK promoter. Furthermore, FOXC1 (WT) was more efficient that FOXC1(p.S131L) in luciferase transactivation showing the importance of functional FOXC1 binding to the upstream SNAP25 region for transactivation. However, no change in FOXC1 ability to transactivate luciferase was observed following deletion of the predicted FOXC1 binding site. It is likely that, as was observed with RAB3GAP1, FOXC1 interaction with the upstream SNAP25 region is not dependent on a single binding site or that it may bind an accessory protein or transcription factor to carry out this increase in transactivation. Though the increase of luciferase expression compares to the mutant (p.S131L) construct indicates that FOXC1 is both sufficient and necessary to cause this transactivation. FOXC1 was able to increase luciferase transactivation through the upstream SNAP25 region only in the presence of the TK promoter. Therefore, the FOXC1 binding site is not located in a promoter region but rather in an enhancer region located ~ 5 kb away from the SNAP25 TSS, similarly to RAB3GAP2.

In HeLa cells we showed that FOXC1 knockdown resulted in decreased RNA levels of both isoforms of *SNAP25* (*SNAP25a* and *SNAP25b*) and reduced SNAP25 protein levels. Interestingly, FOXC1 over-expression in HeLa cells decreased protein levels of SNAP25. A possible explanation is that SNAP25 is subjected to bimodular regulation by FOXC1, where deviations from normal FOXC1 activity ranges, whether above or below a specific threshold, lead to decrease in SNAP25 expression. In our transactivation experiment FOXC1 increased luciferase activation through an upstream SNAP25 when combined with a basal promoter, conflicting the decrease in SNAP25 protein observed with FOXC1 over-expression. However, we cloned a 151 bp region in the transactivation experiment, while in the cell FOXC1 likely interacts with multiple regions upstream of SNAP25 and other co-factors leading to complex bimodular regulation. ARS patients who have missense and frameshift mutations, resulting in reduced FOXC1 function, have similar phenotype to patients with FOXC1 duplications indication a potential role for bimodular regulation of FOXC1 targets in ARS pathogenesis. FOXC1 dosage is crucial since mutations that decrease FOXC1 activity below 80%, and FOXC1 duplications that increase FOXC1 copies to 150% both lead to ARS, with elevated IOP and glaucoma pathogenesis.[43]

SNAP25 has been shown to be essential in glutamatergic and GABAergic neurons for fast neurotransmission in both excitatory and inhibitory circuits [44]. SNAP25 inhibition was also shown to interfere with the formation of the SNARE complex and decreased evoked glutamate release rat cerebellar neurons [45]. Therefore, FOXC1 is likely to play a role in glutamate excitotoxicity through its regulation of SNAP25. SNAP25 is also essential for endocytosis as SNAP25 cleavage with BoNT inhibited endocytosis [46].

Our results indicate that FOXC1 regulates the RAB3GAP complex and SNAP25, both of which have central roles in exocytosis and endocytosis. To determine whether this regulation has physiological consequences, the effect of FOXC1 on MYOC secretion was examined. MYOC is a protein associated with early onset glaucoma, similarly to FOXC1, and is also involved in steroid induced glaucoma [47,48].

Decrease in RAB3GAP and SNAP25 activity has been previously linked to inhibition of exocytosis, and we have shown that FOXC1 knockdown decreases levels of the RAB3GAP subunits, RAB3GAP1 and RAB3GAP2, as well as SNAP25. FOXC1 knockdown would therefore be predicted to inhibit secretion of MYOC. However, FOXC1 knockdown had an unexpected effect on MYOC secretion. Knockdown of FOXC1 or its targets RAB3GAP1, RAB3GAP2 and SNAP25, separately or in combination, lead to a decrease in intracellular MYOC levels. In turn, knockdown of either RAB3GAP1 or SNAP25 resulted in increased extracellular MYOC levels. It is, therefore, likely that knockdown of FOXC1 or its target genes leads to an initial increase in MYOC secretion. However, when RAB3GAP2 is knocked down, there is a reduction in extracellular MYOC, potentially through a process of degradation triggered by RAB3GAP2. MYOC secretion when all three target genes are knocked down simultaneously paralleled the condition in which FOXC1 is knocked down. These findings support the conclusion FOXC1 regulates MYOC secretion through FOXC1’s regulation of RAB3GAP1, RAB3GAP2 and SNAP25. The effect of SNAP25 knockdown on MYOC secretion is especially interesting as SNAP25 is considered to act mainly in neuronal cells while in non-neuronal cells SNAP25 is thought to be replaced by its non-neuronal homolog, SNAP23 [49]. Our results show that SNAP25 is involved in secretion in HeLa cells and might potentially play an important role in other non-neuronal cell lines.

The new connection between MYOC and FOXC1 can improve our understanding of the role of FOXC1 in glaucoma pathogenesis. However, further validation of the interaction between FOXC1 and MYOC in animal models of glaucoma and ocular cell lines would elucidate weather the interaction is specific to HeLa cells or also occurs in tissues affected during glaucoma pathogenesis. Pathogenic MYOC mutations found in glaucoma patients affect MYOC secretion and result in intracellular accumulation of MYOC [29,50]. MYOC accumulation is linked to ER stress and inflammatory stress responses in trabecular meshwork cells [51–53]. There are also increasing evidence pointing to MYOC as an inhibitor of neuronal regeneration. Increased levels of MYOC mRNA and protein were found in glial scars of astrocytes isolated from injured cerebral cortexes compared to uninjured controls [54]. Furthermore, in an assay measuring neurite outgrowth, ganglia stopped neurite outgrowth upon reaching areas with immobilized MYOC protein [54]. These findings indicate a pathogenic mechanism where MYOC acts to prevent regeneration of retinal astrocytes following injury further accelerating their death [55]. Therefore, FOXC1 mutations would likely affect both stress response in the trabecular meshwork and RGC regeneration through MYOC. This novel interaction shows an additional role for FOXC1 in later onset glaucoma.

In conclusion, we have shown that FOXC1 regulates both components of the RAB3GAP complex and SNAP25, linking FOXC1 to regulation of exocytosis. We have also shown that FOXC1 is able to modulate extracellular trafficking of MYOC by controlling levels of the RAB3GAP complex and SNAP25. Our results therefore indicate that ARS patients could be affected by similar glaucoma pathogenic pathways as JOAG patients with MYOC mutations. FOXC1 regulation of exocytosis could also impact ATP release in trabecular meshwork cells, which is important for mobilization of arachidonic acid which in turn is necessary for prostaglandin production [37]. FOXC1 mutations can affect ATP release in the TM and interrupt prostaglandin secretion, which could underlie, in part, the lack of response of ARS patients to IOP lowering prostaglandin medications [10]. In addition, FOXC1 is likely to affect autophagy through its regulation of RAB3GAP. RAB3GAP is associated with autophagosome biogenesis and through its regulation of RAB3 with transferrin receptor degradation leading to autophagic death [39,56]. Thus, through its regulation of RAB3GAP, FOXC1 could be be involved in RGC autophagy induced apoptosis [57]. These findings provide an interesting avenue of research for ARS as an important model of glaucoma, and reveal that FOXC1 mutations are involved in multiple pathways, both in developmental and potentially later onset glaucoma pathogenesis.

## Methods

### FOXC1 binding sites and primers

FOXC1 binding sites (BS) located within 10,000 bp upstream of transcription start sites of target genes were detected by the Possum software (http://zlab.bu.edu/~mfrith/possum/) using a FOXC1 binding matrix [58]. Primers corresponding to predicted FOXC1 binding sites upstream of RAB3GAP1, RAB3GAP2 and SNAP25 were designed using Primer3 (http://bioinfo.ut.ee/primer3-0.4.0/). Primers are described in S1 Table.

### Chromatin immunoprecipitation (ChIP)

HeLa cells were obtained from ATCC (ATCC Number:CCL-2) and grown to confluency then fixed with 1% formaldehyde for 10 minutes and molecular crosslinking was quenched in 1.25 mM glycine for 5 minutes. Media were aspirated and cells washed with PBS (Na_2_PO_4_ 9.1 mM, NaH_2_PO_4_ 1.7 mM, NaCl 150 mM), then lysed with ChIP cell lysis buffer (PIPES pH 8.0 5 mM, KCl 85 mM, IGEPAL CA-630 0.5%) with 1 mM PMSF and 0.05% final concentration of Protease Inhibitor Cocktail (PIC) (Sigma-Aldrich, St. Louis, Missouri, United States) and ChIP Nuclear lysis buffer (Tris pH 8.0 50 mM, EDTA 10 mM, SDS 1%) with 0.05% PIC and 1 mM PMSF with the aid of a Dounce homogenizer. Nuclear lysates were sonicated with 3x 15 seconds pulses (5–7 W) using a Sonic dismembrator 60 (Thermo Fisher Scientific, Waltham, Massachusetts, United States) on ice, with 15 seconds of rest between each pulse. Protein A/G PLUS-Agarose (Santa Cruz Biotechnology, Dallas, Texas, United States) beads and 10 mg/ml sonicated salmon sperm ssDNA (Invitrogen, Carlsbad, California, United States) were added to lysates and diluted with ChIP Dilution Buffer (Tris pH8.0 16.7 mM, EDTA 1.2 mM, NaCl 167 mM, SDS 0.01%, Triton X-100 1.1%, PIC 0.05%, 1 mM PMSF). Three µg of either Rabbit anti-IgG (CALTAG Laboratories Buckingham UK), a negative control, Rabbit anti-H3K4ME3 (a generous gift from Dr. David Eisenstat, University of Alberta, Edmonton), a positive control for transcriptionally active chromatin, or Goat anti-FOXC1(OriGene, Rockville, Maryland, United States) were used for each immunoprecipitation reaction. Beads were washed with ChIP Wash Buffer (Tris 20 mM, EDTA 2 mM, SDS 0.1%, Triton X-100 1%), ChIP Final Wash Buffer (Tris pH 8.0, EDTA 2 mM, NaCl 500 mM, SDS 0.1%, Triton X-100 1%), LiCl wash buffer (0.25 M LiCl, 1% deoxycholate, 1 mM EDTA, 10 mM Tris-HCl (pH 8.1), 1% NP-40) and TE buffer pH8.0 (2x). The DNA was eluted with ChIP Elution Buffer (Sodium Bicarbonate 100 mM, SDS 1%) and purified with a QIAGEN quick PCR Purification kit (Qiagen, Hilden, Germany), according to the manufacturer’s protocol. One µl of purified DNA was used as a template for amplified with PCR using ChIP primers for *RAB3GAP1*, *RAB3GAP2* and *SNAP25* described in S1 Table, then used for DNA electrophoresis on 1% agarose gel stained with SYBR® Safe (ThermoFisher Scientific, Waltham, Massachusetts, United States). Images were taken with Imagestation4000 after a 1minute exposure. Quantitative PCR analysis was performed to verify the results of the qualitative agarose gel analysis. q-PCR assays were performed with the QuantiTect SYBR Green PCR kit (Applied Biosystems Foster City, California, United States). The fold enrichment method (Primer efficiency to the power of ΔCt [Ct_Target_-Ct_IgG]_)) was used to determined enrichment of RAB3GAP1.BS1, RAB3GAP2.BS2, and SNAP25.BS1 upon immunoprecipitation with the above FOXC1 antibody. Primer efficiencies were calculated using the standard curve method.

### Expression constructs

pcDNA4:Xpress-Empty vector was purchased from Invitrogen. pcDNA4:Xpress-FOXC1(WT) was created as described previously [16]. A pcDNA4:Xpress-FOXC1 (p.S131L) construct, containing a patient mutation resulting in reduced binding of FOXC1 to its target genes was created and used, as described previously[16].

### Luciferase transactivation assay

To clone upstream target gene regions containing predicted binding sites into pGL3 and pGL3.TK, SacI and BglII binding sites were added to the RAB3GAP1, RAB3GAP2, SNAP25 ChIP primers as described in S1 Table. PCR products were cloned into pGL3 (Promega Madison, Wisconsin, United States) to generate the plasmid constructs pGL3.RAB3GAP1, pGL3.RAB3GAP2 and pGL3.SNAP25. Additionally, upstream RAB3GAP2 and SNAP25 regions were cloned into the pGL3.TK reporter, which contains the proximal promoter region of the TK gene[16], to generate pGL3.TK.RAB3GAP2 and pGL3.TK.SNAP25 constructs, respectively. Additional constructs were generated with the predicted binding sites deleted using site directed mutagenesis (QuickChange Lightning Kit, Agilent technologies Santa Clara, California, United States) with the primers described in S1 Table. The RAB3GAP1 upstream region contained three potential binding sites and each was deleted separately and in all possible combinations to generate seven constructs: pGL3-Del BS1, pGL3-Del BS2, pGL3-Del BS3, pGL3-Del BS1+2, pGL3-Del BS1+3, pGL3-Del BS2+3, pGL3-Del1+2+3. In each of the RAB3GAP2 and SNAP25 upstream regions, one predicted FOXC1 binding site was deleted to generate: pGL3.RAB3GAP2.del, pGL3.TK.RAB3GAP2.del, pGL3.SNAP25.del, and pGL3.TK.SNAP25.del. The FOXC1 responsive plasmids pGL3.FOXO1 (containing the FOXC1 responsive element of the FOXO1A promoter) and pGL3.TK.6xFBS (containing cloning six copies of the FOXC1-binding site) were used as a positive controls for Luciferase transactivation as described previously[19]., Plasmids were used in HeLa transfections using Lipofectamine2000® reagent (Invitrogen Carlsbad California, United States) in accordance with the manufacturer’s instructions. Cells within a 24 well plate (4.0x10^5^ cells/well) were transfected in triplicate with 30 ng pRL-CMVβ (Promega) as a transfection efficiency control and either 500 ng pcDN4:Xpress-FOXC1 (WT) or pcDNA4:Xpress-FOXC1 (p.S131L), to compare the ability of WT versus mutant FOXC1 to activate the Luciferase reporter.

Three days after transfection, Luciferase transactivation assays were performed using the Promega Dual–Luciferase reporter assay system and the β-Galactosidase Enzyme Assay System per the manufacturer’s protocol. Luciferase values were normalized to β -galactosidase values to control for transfection efficiency, then scaled to the activation level of the empty Luciferase reporter when treated with pcDNA4:Xpress-FOXC1(WT) to compare fold change in activation.

### Transfections for qRT-PCR and Western analysis

HeLa cells were transfected using the Lipofectamine2000® reagent (Invitrogen), per the manufacturer’s instructions. For FOXC1 knockdown experiments, 500 ng of Silencer negative control siRNA #1 (Ambion, Thermo Fisher Scientific) or FOXC1 siGENOME #4 (Dharmacon Lafayette, Colorado, United States) were used. For FOXC1 over-expression experiments, 12.5 ug of either pcDNA4:Xpress-Empty or pcDNA4:Xpress- FOXC1 (WT) were used. The transfected cells were grown for 72 hours in 37°C and 5% CO_2_ prior to collection.

### qRT-PCR

Cells were washed twice with PBS, and then treated with 1ml Trizol (Ambion) to isolate total RNA. Two μg of RNA were used for cDNA preparations with M-MLV Reverse Transcriptase (Invitrogen) according to the manufacturer’s protocol (Invitrogen).

Primers for *FOXC1*, *HPRT1* (a housekeeping gene, used as a reference gene for normalization) *RAB3GAP1*, *RAB3GAP2* and *SNAP25* (detecting both isoforms) were designed using the Primer3 software. Both isoforms of *SNAP25* were examined as they may be regulated through different transcriptional pathways. Primers were designed to separately detect the *SNAP25a* and *SNAP25b* isoforms as described previously [59]. The primer sets are described in S1 Table. qRT-PCR assays were performed with a QuantiTect SYBR Green PCR kit (Applied Biosystems Foster City, California, United States) and run on a 7900HT Fast Real-Time PCR System (Applied Biosystems) at least three times with each reaction in triplicate. Dissociation curves were performed with each qRT-PCR to confirm the homogeneity of the PCR product and primer specificity. RNA levels were normalized to *HPRT1* through the ΔΔCt method and changes in RNA levels were described in fold change compared to the control treatment.

### Western blot analysis

HeLa cells were lysed with IP lysis buffer (IGEPAL ® CA-680, Tris pH 8.0 0.05 M, NaCl 0.15 M, PMSF 1 mM, Protease Inhibitor Cocktail 0.05%), and denatured with for 10 minutes at 65°C with 6x SDS-PAGE Loading Buffer (Stacking Gel Buffer -0.06 M Tris 0.05% SDS, Glycerol 10%, SDS 0.02%, DTT 1%, Bromophenol Blue 0.05%), then size separated on 8% SDS-PAGE and transferred to a nitrocellulose membrane (Bio-Rad). Blots were incubated overnight at 4°C with either of the following primary antibodies: Rabbit anti-FOXC1 (Cell Signaling, ‎Danvers, Massachusetts, United States) 1:5000, Rabbit anti-RAB3GAP1 (AssayBioTech Sunnyvale, California, United States) 1:5000, Rabbit anti-RAB3GAP2 (AssayBioTech) 1:5000, Rabbit anti-SNAP25 (Abcam, Cambridge, United Kingdom) 1:1000 in 5% skim milk in TBST. Blots were also incubated with mouse anti- α-Tubulin (Santa Cruz Biotechnology), as loading control, diluted 1:5000 in 5% skim milk in TBST and a secondary HRP-conjugated antibody diluted 1:5000 in 5% skim milk in TBST for 1 hour in room temperature. Secondary antibody signal was exposed by SuperSignal™ West Femto Maximum Sensitivity Substrate (Thermo Scientific). Images were taken on Imagestation4000 with 10-minute exposure. Net intensity of bands was normalized to the net intensity of α-Tubulin then scaled to the control for each treatment. Experiments were repeated at least in triplicate.

### Analysis of Myocilin secretion following FOXC1 knockdown

HeLa cells were transfected using Lipofectamine2000® with 12.5 µg of pRc-Myocilin-Myc[29] and either 500 ng of Silencer negative control siRNA#1 or FOXC1 siGENOME #4 (siRNA FOXC1). After 72 hours a 1 ml sample of the cell media was collected and cells were lysed with IP Lysis buffer as described in earlier sections. MYOC and FOXC1 levels were analyzed by western blot analysis using Rabbit anti-FOXC1 and Goat anti-MYOC (Santa Cruz Biotechnology). The MYOC band net intensity was normalized to Ponceau Red stain (total protein control) net intensity in media bands. Whole cell lysates were normalized to the loading control, TFIID, after incubation for 1 hour in room temperature with 1:5000 Rabbit anti-TFIID (Santa Cruz Biotechnology). After normalization protein levels were scaled to the treatment control. Experiments were repeated in triplicate.

### Analysis of Myocilin secretion following RAB3GAP1, RAB3GAP2 and SNAP25 knockdown

HeLa cells were transfected using Lipofectamine2000® with 12.5 µg of pRc-Myocilin-Myc and either 250 ng of Silencer negative control siRNA #1, siRNA RAB3GAP1(5´-UCAGUACACUCACUUAUCA-3´), siRNA RAB3GAP2 (5´-UGACUUGGCUCUGUUACUA-3´), both custom ordered from Dharmacon and designed after Spang et al. (2014)[42], a combination of siRNA RAB3GAP1 and siRNA RAB3GAP 2, SNAP25 Silencer® Select S13188 (Ambion, Thermo Fisher), and a combination of RAB3GAP1, siRNA RAB3GAP2 and SNAP25 Silencer® Select. After 72 hours, media samples were collected and cells were lysed for western blot analysis, then whole cell and media MYOC protein levels were measured as described previously. Blots were then incubated with either Rabbit anti-RAB3GAP1 (1:5000), Rabbit anti-RAB3GAP2 (1:5000) or Rabbit anti-SNAP25 (1:1000) overnight in 4°C and for an hour the following day with their secondary HRP-conjugated antibody diluted 1:5000 in 5% skim milk. Protein net intensity was normalized to TFIID and scaled to the treatment control for each experiment to examine fold change. Experiments were repeated in triplicate.

### Statistical analysis

Statistical significance was evaluated using the Mann-Whitney U test. P-values were calculated using AI-Therapy Statistics software (https://www.ai-therapy.com/psychology-statistics/hypothesis-testing/two-samples). One-tailed analysis was carried out to confirm *FOXC1*, *RAB3GAP1*, *RAB3GAP2* and *SNAP25* knockdown with siRNAs and FOXC1 over-expression with pcDNA4:Xpress-FOXC1 (WT). ChIP-qPCR analysis was performed using a one-tailed Mann Whitney U-test. One tailed analysis was employed when the directionality of the experiment was known (i.e. knockdown of a gene, enrichment of binding in ChIP). For experiments where directionality was not known two-tailed analysis was used. Correlation of increase in RAB3GAP2 protein levels with increase in FOXC1 protein levels was evaluated using the non-parametric Spearman's-Rho test with the Social Sciences Statistics software (http://www.socscistatistics.com/tests/spearman/Default2.aspx).

## Supporting information

S1 Fig Luciferase transactivation by FOXC1 through upstream regions of *RAB3GAP2* and *SNAP25* in a pGL3 reporter. Transactivation experiments with plasmid expressing FOXC1, and reporter construct (pGL3) containing **(A)** The 211bp *RAB3GAP2* upstream region, pGL3.R3G2.del and pGL3.FOXO1 (positive control) **(B)** The 151bp *SNAP25* upstream region (pGL3.SNAP25) and SNAP25.del. All Experiments were repeated at least three times in triplicate. Error bars represent standard error. N.S Not significant, *P˂0.05, versus pGL3.
(PDF)

S2 Fig Endogenous and exogenous MYOC levels in HeLa cells. HeLa cells were either transfected with pRc-MYOC (WT) or left untreated. After three days a sample of cell media was taken and the cells were lysed. Protein lysates or media samples were used for western blot analysis. Antibodies against MYOC and TFIID (loading control) were used to detect proteins. Ponceau red stain was used for media sample total protein control.
(PDF)

S1 Table Primers used for cloning and PCR. (PDF)
