# Supplementary figures and images for: FOXC1 modulates MYOC secretion through regulation of the exocytic proteins RAB3GAP1, RAB3GAP2 and SNAP25
Source: PLoS One. 2017 Jun 2;12(6):e0178518. doi: 10.1371/journal.pone.0178518 (PMC5456087; doi:10.1371/journal.pone.0178518)

## Supporting information

Fig S1.

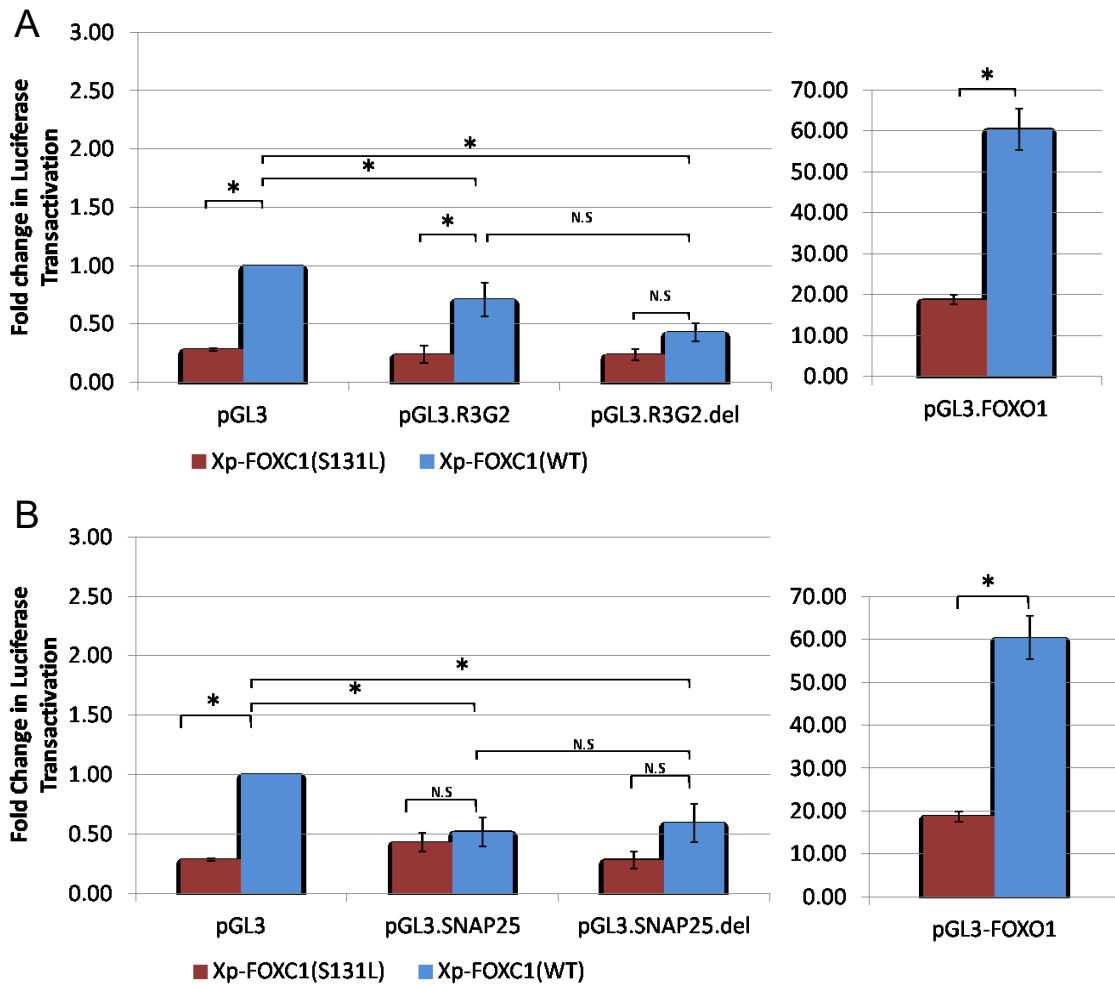

Supplement: S1 Fig — Transactivation experiments with plasmid expressing FOXC1, and reporter construct (pGL3) containing (A) The 211bp RAB3GAP2 upstream region, pGL3.R3G2.del and pGL3.FOXO1 (positive control) (B) The 151bp SNAP25 upstream region (pGL3.SNAP25) and SNAP25.del. All Experiments were repeated at least three times in triplicate. Error bars represent standard error. N.S Not significant, *P˂0.05, versus pGL3. (PDF) [file pone.0178518.s001.pdf]

**Fig S2.****Untreated pRcMYOC**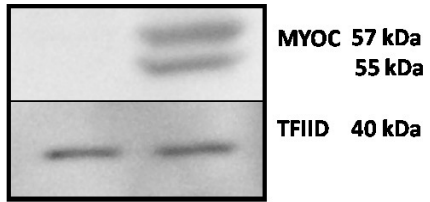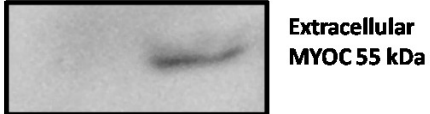

Supplement: S2 Fig — HeLa cells were either transfected with pRc-MYOC (WT) or left untreated. After three days a sample of cell media was taken and the cells were lysed. Protein lysates or media samples were used for western blot analysis. Antibodies against MYOC and TFIID (loading control) were used to detect proteins. Ponceau red stain was used for media sample total protein control. (PDF) [file pone.0178518.s002.pdf]
