# Supplementary material for: FOXC1 modulates MYOC secretion through regulation of the exocytic proteins RAB3GAP1, RAB3GAP2 and SNAP25
Source: PLoS One. 2017 Jun 2;12(6):e0178518. doi: 10.1371/journal.pone.0178518 (PMC5456087; doi:10.1371/journal.pone.0178518)
Supplement: S1 Table — (PDF) [file pone.0178518.s003.pdf]

S1 Table.

Sense and Antisense Primers		
**a. ChIP PCR**	**Sense**	**Antisense**
RAB3GAP1	aggggcagagggtagaagtg	aagggaacagaagccaaggt
RAB3GAP2	caggcaaaatctgcatttcat	tcgagaattaaggagggacaaa
SNAP25	aggggtaagtacatgccgtc	catcacacttctgccagctc
**b. Transactivation cloning**	**Sense**	**Antisense**
RAB3GAP1	gagctcaggggcagagggtagaagtg	agatctaagggaacagaagccaaggt
RAB3GAP2	agatctcaggcaaaatctgcatttcat	gagctctcgagaattaaggagggacaaa
SNAP25	gagctcaggggtaagtacatgccgtc	agatctcatcacacttctgccagctc
**c. Mutagenesis**	**Sense**	**Antisense**
***RAB3GAP1*.del1 (129-144)**	ctaattggtactaactacaaatatgggtcaacatgctttaggttttttaatacaat	attgtattaaaaaacctaaagcatgttgacccatatttgtagttagtaccaattag
***RAB3GAP1*.del2 (35-50)**	gtagaagtggtattacagcacctatcaataaatagagggtgaatga	tcattcaccctctatttattgataggtgctgtaataccacttctac
***RAB3GAP1*.del3 (67-82)**	tgccttacaatcaataaatagagggcaatagttaaagtgtaagctaattg	caattagcttacactttaactattgccctctatttattgattgtaaggca
***RAB3GAP2*.del (54-70)**	caggagagaggaatagtctgtgcccttgagtcatttg	caaatgactcaagggcacagactattcctctctcctg
***SNAP25*.del (165-180)**	gtcatggtccaaaggacttttcaggttcacctgac	gtcaggtgaacctgaaaagtcctttggaccatgac
**d. qRT-PCR**	**Sense**	**Antisense**
SNAP25ab	gtgtagtggacgaacgggag	ccatatccagggccatgtgac
SNAP25a	aagggctgaccagttggctgatgagt	ttggttgatatggttcatgccttcttcgacacga
SNAP25b	aagggctgaccagttggctgatgagt	cttattgatttggtccatcccttcctcaatgcgt
RAB3GAP1	aagccaagtttggaactgagaa	ctgcaccggtgactaacactt
RAB3GAP2	ctccacacccagacaaaagaa	gcaattggcacttcagtgttt
FOXC1	tagctgtcaaatggccttccc	cttttcctgcttttggggttcg
HPRT1	gccagactttgttggatttga	ggctttgtattttgcttttccag
STXBP6	attaggccgagcagaggagaa	agaagagtcaccaggataggca
SNAP23	tgagtctctggaaagtacgagga	tccaagccttcttctatgcgg
SYN2	acaggtacaaactgtgggtgg	atcagtggcatgctacagtcc
